# Clinical Priority Setting and Decision-Making in Sweden: A Cross-sectional Survey Among Physicians

**DOI:** 10.34172/ijhpm.2021.16

**Published:** 2021-03-15

**Authors:** Catharina Drees, Barbro Krevers, Niklas Ekerstad, Annette Rogge, Christoph Borzikowsky, Stuart McLennan, Alena M. Buyx

**Affiliations:** ^1^Division of Biomedical Ethics, Institute of Experimental Medicine, ChristianAlbrechts-University of Kiel, Kiel, Germany.; ^2^Department of Health, Medicine and Caring Sciences, Unit of Health Care Analysis, Linköping University, Linköping, Sweden.; ^3^National Centre for Priorities in Health, Linköping University, Linköping, Sweden.; ^4^NU Hospital Group, The Research and Development Unit, Trollhättan, Sweden.; ^5^Institute of Medical Informatics and Statistics, University Hospital Schleswig-Holstein, Kiel, Germany.; ^6^Institute of History and Ethics in Medicine, Technical University of Munich, Munich, Germany.; ^7^Institute for Biomedical Ethics, University of Basel, Basel, Switzerland.

**Keywords:** Priority Setting, Sweden, National Guidelines, Physician, Fair Allocation, Decision-Making

## Abstract

**Background:** Priority setting in healthcare that aims to achieve a fair and efficient allocation of limited resources is a worldwide challenge. Sweden has developed a sophisticated approach. Still, there is a need for a more detailed insight on how measures permeate clinical life. This study aimed to assess physicians’ views regarding (1) impact of scarce resources on patient care, (2) clinical decision-making, and (3) the ethical platform and national guidelines for healthcare by the National Board of Health and Welfare (NBHW).

**Methods:** An online cross-sectional questionnaire was sent to two groups in Sweden, 2016 and 2017. Group 1 represented 331 physicians from different departments at one University hospital and group 2 consisted of 923 members of the Society of Cardiology.

**Results:** Overall, a 26% (328/1254) response rate was achieved, 49% in group 1 (162/331), 18% in group 2 (166/923). Scarcity of resources was perceived by 59% more often than ‘at least once per month,’ whilst 60% felt less than ‘well-prepared’ to address this issue. Guidelines in general had a lot of influence and 19% perceived them as limiting decision-making. 86% professed to be mostly independent in decision-making. 36% knew the ethical platform ‘well’ and ‘very well’ and 64% NBHW’s national guidelines. 57% expressed a wish for further knowledge and training regarding the ethical platform and 51% for support in applying NBHW’s national guidelines.

**Conclusion:** There was a need for more support to deal with scarcity of resources and for increased knowledge about the ethical platform and NBHW’s national guidelines. Independence in clinical decision-making was perceived as high and guidelines in general as important. Priority setting as one potential pathway to fair and transparent decision-making should be highlighted more in Swedish clinical settings, with special emphasis on the ethical platform.

## Background

**Key Messages**

** Implications for policy makers**

- In the pursuit of fair allocation of scarce healthcare resources the Swedish approach to priority setting is an instructive example. Physicians in Sweden overall do not feel restricted in their independent clinical decision-making by national priority-setting guidelines.
- Firm and well-reasoned guidelines are important for clinical decision-making and appreciated by clinicians.
- Continuous communication on priority setting guidance at all levels of experience, as well as training and support, are needed and should be further developed and established.
- The implementation of ethical priority setting can be seen as one pathway towards higher equity and quality in healthcare by enabling transparent decision-making.

** Implications for the public**

Resources in healthcare will never be unlimited. Rising demands and rising diagnostic and treatment possibilities are continuously increasing costs. This has long been recognized as a challenge both for health policy-makers as well as for physicians. Decision-making about scarce resources includes difficult questions about fairness, need, equity, solidarity etc. Priority setting is one option to address this challenge. It combines ethical principles and transparent discussions to enable fair and evidence-based guidance on resource allocation. Sweden’s approach to priority setting has been widely recognized as an early and successful approach. This study investigated how Swedish physicians view their country’s priority setting approach and its impact on their clinical decision-making. It thus provides important findings on how a well-established priority setting approach translates into the clinic, and affects those who have to implement it, thereby offering an instructive example for other countries struggling with questions of fairness and equity in healthcare allocation.

Healthcare is essential in all countries; promoting not only individual health and well-being but also employment, economic growth, social stability, health security, social cohesion, political stability, and economic diversification and innovation.^1,2^ However, healthcare systems around the world are increasingly facing challenges with regard to rising costs; with health costs increasing faster than the total economy in most countries.^3^ Priority setting, in addition to rationing and rationalisation, is one of the key approaches in addressing these challenges. This involves deciding on the relative importance of services.^4^ The process of resource allocation within healthcare involves many different stakeholders. While the macro level, consisting of national, regional and/or supranational policy-making, sets the framework, and the meso level involves the institutional level of hospitals, it is individual physicians (or other healthcare professionals) on the micro level who ultimately make the final clinical priority decisions on a daily basis.^5^ Physicians need to treat patients based on the evidence and offer what is medically indicated, as well as cost effective. This phenomenon has been described as the ‘double agent’ phenomenon.^6-8^ Against a background of scarcity, priority setting guidance at the clinical level can serve to solve potential conflicts between cost-effectiveness and medical indication, and enable transparent discussions about allocation of resources.

 Sweden’s approach to priority setting in healthcare is recognised as one of the early and most meaningful approaches internationally, with elaborated methods of implementation for a fair resource allocation system.^8-10^ Although Sweden’s healthcare system is led by national government policies, the system is highly decentralised. The coverage is universal, automatic and tax-funded by regions and municipalities. Fully privately funded care is rare, but in addition to publicly funded healthcare, about 10% of all Swedish employees have supplementary private health insurances.^11,12^ Priority setting in Sweden became recognised in 1992 when the Swedish government initiated a Parliamentary Priorities Commission consisting of politicians and professional experts. A parliamentary decision in 1997 ratified the Commission’s proposal and the Health and Medical Services Act was amended accordingly.^13^ The primary outcome of this decision was the ethical platform consisting of three key ethical principles in descending order: (1) Principle of human dignity: ensuring equal human values and rights, (2) Principle of need and solidarity: ensuring distribution according to the greatest needs, (3) Principle of cost-effectiveness: ensuring reasonability between costs and effects.^14^

 The Swedish National Board of Health and Welfare (NBHW) has developed national guidelines in collaboration with experts and healthcare professionals, to function as support in priority setting for decision-makers in healthcare. They combine the currently best available evidence – including, if available, cost-effectiveness data – with the recommendation of priority setting based on the ethical principles. They can be regarded as a type of knowledge governance in the pursuit of equal and effective healthcare. They include non-binding recommendations in the form of rankings of condition and treatment pairs, ranked from one to ten, with one marking the highest and ten the lowest priority. In 2004, the first national guideline by NBHW was published in the field of cardiac care. By 2021, 18 different NBHW national guidelines for specific disease groups had been published. The combination of ethical aspects, best medical evidence and cost-effectiveness data sets the NBHW’s national guidelines apart from most other clinical guidelines, be they international, national, or local ones. The ethical platform has remained unchanged to the present day and serves as the core foundation of all future measures of priority setting.

 To date, there have only been a few studies examining physicians’ perception of clinical priority setting in Sweden. Most of them were published shortly after the ethical platform for priority setting was introduced and the focus was on cardiology, as the first NBHW’s national guideline concerned cardiology.In 2003, it was reported that 64% of clinicians were informed about priority setting, 40% to 55% knew the meaning of each ethical principle and 58% knew the national guideline for coronary artery diseases, a predecessor of the later and broader national guideline for cardiac care.^15^ In 2010, 90% of clinicians were reported to be very familiar with the contents of the national guideline on cardiac care and 81% used it extensively.^16^ However, in 2013, it was reported that only a small amount of personnel in general practices knew about the ethical principles and the NBHW’s national guidelines and that they were unsure about the contents.^17^ In 2015, it was reported that 76% of the heads of different hospital departments applied the NBHW’s national guidelines, 33% in their original form, 69% through local guidelines.^18^ Further studies have reported that physicians expressed a lack of support structures^19^ and a lack of basic training and support of ethical principles.^15^ There was a need for more open dialogues and discussions about priority setting, more practical and clearer national guidelines,^15,19^ as well as more evidence for the treatment of elderly multimorbid patients.^20^

 Although Sweden has developed a sophisticated approach to priority setting in healthcare, there is thus still a need for a more detailed insight on how it permeates the micro level of decision-making. Most of the former research focused on specific aspects of the available guidance, but a wider perspective is needed to evaluate the existing priority setting measures. To date, studies focussing on how the national priority setting approach is perceived by physicians in clinical decision-making are lacking. Such research requires an understanding of the situation of physicians, including their background of patient care and decision-making. This study therefore included such information; it aimed to examine physicians’ views regarding (1) the impact of scarce resources on patient care, (2) priority setting and clinical decision-making, and (3) the ethical platform and the NBHW’s national guidelines for healthcare including recommendations for priority setting. These were investigated in a local sample in one large healthcare institution across different departments (group 1) and in a national sample of physicians in the field of Cardiology, where priority setting guidance exist longest (group 2). This was done to achieve local depth across various specialties, as well as spread across the country.

## Methods

 The study did not require approval by a research ethics committee in Sweden, according to the Swedish legislation.^21^ However, the ethical principles of the declaration of Helsinki were observed throughout.^22^

###  Survey Implementation and Population

 The online survey was conducted between August 2016 and January 2017. Two samples were generated (N in total = 1254). The first sample consisted of 331 physicians from twelve different departments at the Örebro University hospital including general internal medicine, cardiology, pulmonology, geriatrics, general surgery, cardiothoracic and vascular surgery, anaesthesia/intensive care, emergency department, urology, oncology, neurology, and psychiatry. Departments were chosen based on the existence of corresponding national guidelines. The second sample consisted of 923 members of the Swedish Society of Cardiology (predominantly clinical cardiologists). Swedish invitation emails were sent, including information about the aim of the study and Swedish priority setting as well as a personal transaction link to the English online questionnaire, created with the EvaSys automation software.^23^ Reminders were sent up to four times over several weeks and reasons were asked in the event of non-participation. Due to the setting options of the EvaSys automation software^23^ no missing values occurred.

###  Survey Contents

 The survey was based on a questionnaire in English language, created by the researchers. It was based on an extensive literature review, established methods of questionnaire development,^24,25^ comparisons to similar questionnaire studies^15,20,26,27^ and discussions with many different Swedish and German medical specialists as well as experts at the Swedish National Centre for Priorities in Health Care. The questionnaire consisted of 46 questions in total and was pilot tested in Germany as well as in Sweden, taking approximately 15 to 30 minutes to complete. It was divided into three parts; the selection of questions and response options was again based on literature research and aforementioned studies as presented in the introduction section.

 The first part of the survey consisted of general questions about patient care, resources in clinical work, and clinical decision-making. The second part continued with questions about physicians’ awareness and application of the ethical platform and the NBHW’s national guidelines. The third part included demographic questions. To clarify which guidelines were meant, the ones including priority setting measures created by NBHW were described as ‘Socialstyrelsen’s national guidelines’ in the questionnaire, in order to distinguish these from other national and international guidelines not including priority setting measures. The specificity of the NBHW’s national guidelines regarding priority setting was stressed twice, once in the invitation letter and once in the questionnaire, to prevent false responses. The questionnaire strove for simple, descriptive language, therefore no supplement with further explanations was added.

 The most common type of answering scale was an ordinal-scaled five-point Likert scale with responses ranging from either 1 = ‘never’ to 5 = ‘very often;’ 1 = ‘not at all’ to 5 = ‘very well’ or 1 = ‘at least once per day,’ 2 = ‘at least once per week,’ 3 = ‘at least once per month,’ 4 = ‘less than once per month’ to 5 = ‘until now never.’ Another common question type were multiple choice questions with either one answer or multiple answers possible. The possibility of answering ‘can’t say’ was provided for each question. Each question needed to be answered in order to finish the survey, with the exception of open-ended questions and participants who reported no knowledge of the ethical platform or of any of the NBHW’s national guidelines. These participants were automatically directed to the next question they were able to answer.

###  Data Analysis

 The data management and analysis of the quantitative results were performed using the IBM SPSS Statistics for Mac version 24.^28^ Descriptive and inferential statistics were calculated.^29,30^ In order to analyse whether a difference existed between the two sample groups, the two-dimensional chi-square-test was used for categorical nominal or ordinal-scaled data including *P* values. The expected frequencies were greater five in each of the response categories and thereby the assumptions of the chi-squaretest were met. The significance level was α = 0.05. A gender analysis was conducted and *P* values were calculated with Fisher exact test. In cases of metric scaled response categories medians, means and standard deviations as well as the two-sample *t* tests for independent samples were calculated for examining differences. Due to the large sample sizes in both groups *t* tests were robust against non-fulfilling the assumption of normal distribution. Levene test was used to check for the assumption of equal variances. Some questions were multiple-choice questions. In these cases, responses to each single response category were coded as 0 = no (disagreement) or 1 = yes (agreement). It was calculated with the cumulative percentages of agreement.

## Results

 The presented results are mainly based on the total sample, including groups 1 and 2. Results that concern group 1 (participants from one University hospital) and group 2 (participants from the national cardiology register) are only stated in case of relevant and significant differences between the two groups. The physicians are called ‘the participants’ or ‘group 1’ or ‘group 2’ from here on in order to avoid misunderstandings. All results can be found in Supplementary file 1.

###  Characteristics of Respondents

 Overall, a total of 328 completed questionnaires were returned (26% response rate). In group 1, 49% of participants completed the survey (162/331). Participants of group 1 who received the invitation email were 60% male. This proportional distribution is in line with the actual participants of group 1, who were 61% male. In group 2, 18% of the members of the Swedish Society of Cardiology completed the survey (166/923). The members were not only clinical physicians. The mean age of participants of group 2 was 49.9 years, and the percentage of male participants was 73.5%. Based on a comparison with demographic data of the society published in another study, where the mean age was 54 years, and 75% male,^31^ the demographic composition of our sample is well representative of cardiologists in Sweden. Gender effects were rarely observed, meaning that overall presented findings are not seriously influenced by participants’ gender. Significant findings are stated. Further demographic data can be seen in Table.

**Table:** Demographic and Work-Related Characteristics

**Demographic and Work Related Characteristics**	**Group 1**	**Group 2**	**Total**	**Test**	***P*** ** Value **
Participants in total	162	166	328		
Average age in years (SD)	44.0 (11.7)	49.9 (11.6)	47.0 (12.0)	t(326) = -4.56	<.00
Average years of clinical work (SD)	16.6 (11.8)	21.5 (10.9)	19.1 (11.6)	t(326) = -3.89	<.00
Gender (%)					
Female	38.9	26.5	32.6	χ^2^(328) = 5.72	.02
Male	61.1	73.5	67.4		
Employment (%)					
Not legitimised assistant doctor	8.0	0.6	4.3	χ^2^(328) = 24.48	<.00
Legitimised assistant doctor	17.9	9.0	13.4		
Specialist	28.4	26.5	27.4		
Senior doctor	40.7	50.6	45.7		
Chief of department	3.7	7.8	5.8		
Other	1.2	3.6	2.4		
No employment	0.0	1.8	0.9		
Specialisation (%) – most common (over 5%) – multiple responses					
None – still under training	19.8	6.6	13.1	χ^2^(328) = 12.40	<.00
Internal medicine	16.0	71.7	44.2	χ^2^(328) = 102.90	<.00
Anaesthesia and intensive care	12.3	0.0	6.1	χ^2^(328) = 21.83	<.00
Surgery	11.1	0.0	5.5	χ^2^(328) = 19.52	<.00
Oncology	8.6	0.0	4.3	χ^2^(328) = 14.99	<.00
Cardiology	7.4	82.5	45.4	χ^2^(328) = 186.64	<.00
Urology	7.4	0.0	3.7	χ^2^(328) = 12.76	<.00
Hospital size (%)					
Small	0.0	16.3	8.2	χ^2^(328) = 110.28	<.00
Mid-sized	0.6	25.9	13.4		
Large/academic	99.4	48.2	73.5		
Other	0.0	9.6	4.9		
Region/landsting (former official name of regional councils) (%) – most common (over 5%)					
Region Örebro län	96.9	0.0	49.4	χ^2^(328) = 293.77	<.00
Stockholms läns landsting	0.0	19.3	9.8		
Västra Götalandsregionen	0.0	16.9	8.5		
Region Skåne	0.0	15.1	7.9		
Region Östergötland	0.0	7.8	4.0		
Landstinget i Uppsala län	0.0	5.4	2.7		
Participation in writing guidelines (%) – multiple responses					
Local guidelines	53.1	57.8	55.5	χ^2^(328) = 0.75	.39
National guidelines of NBHW	13.6	13.9	13.7	χ^2^(328) = 0.01	.94
Other national guidelines than the ones from NBHW	15.4	9.0	12.2	χ^2^(328) = 3.13	.08
National recommendations on internet-based support resources	1.2	4.2	2.7	χ^2^(328) = 2.73	.10
None	39.5	36.1	37.8	χ^2^(328) = 0.39	.53

Abbreviations: SD, standard deviation; NBHW, National Board of Health and Welfare.

###  Clinical Patient Care

 Regarding patient care, 53% of the participants had the impression that all patients were ‘very often’ treated equally. 47% stated that all resources were ‘often’ distributed according to patients’ healthcare need. Seventy-two percent thought that all decisions were ‘somewhat’ (37%) to ‘often’ made considering costs in relation to patients’ benefit and 49% reported that all patient care was ‘often’ optimal. The majority of participants, 59%, faced scarcity of resources more often than ‘at least once per month.’ For 43% scarcity led ‘never’ and for 34% ‘less than once per month’ to a non-prescription or non-performance of a therapeutic or diagnostic measure during the last 6 months before data gathering. About a tenth of the participants, 11%, did not prescribe or perform a medical measure ‘at least once per month,’ as well as, 9%, ‘at least once per week’ during the last six months before the survey. Most participants, 60%, felt less than ‘well’ prepared to handle scarcity of resources.

**Figure 1:**
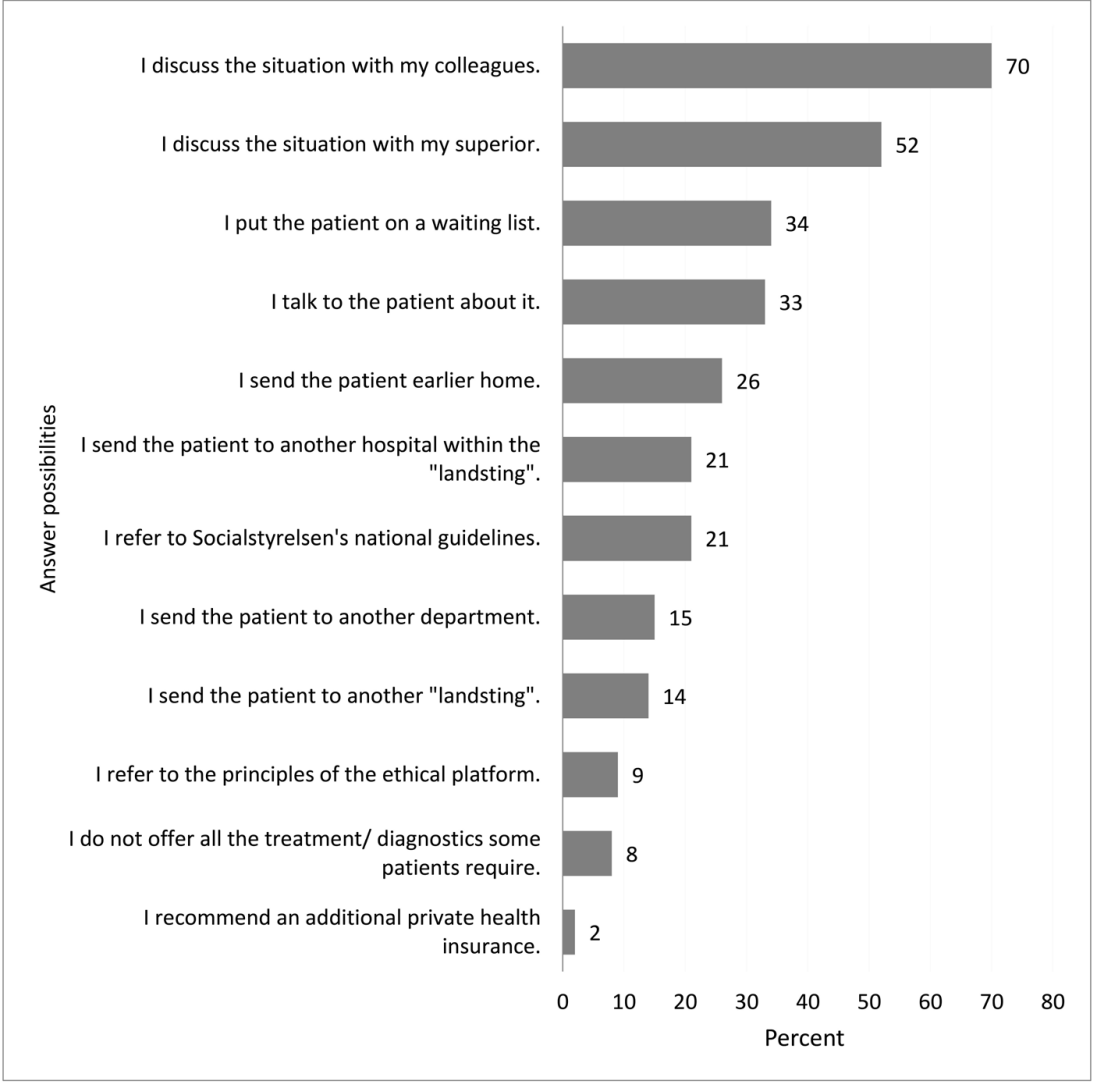
How the Participants Handle Scarcity of Resources in Their Clinical Work (Group 1+2).

 The four most important strategies of dealing with perceived scarcity of resources were: (1) discussing with colleagues – 70%, (2) discussing with superiors – 52%, (3) putting patients on a waiting list – 34%, (4) talking to the patient about it – 33%. Rationing – ‘not offering all the treatment/diagnostic some patients required’ – occurred in 8% of all participants. There was a significant difference in how often participants referred to the NBHW’s national guidelines and the ethical platform, with group 2 referring to these more often than group 1 (national guidelines: group 1: 15%; group 2: 28%; group 1+2: 21%, χ^2^ (328) = 8.12 with *P* < .00; the ethical platform: group 1: 4%; group 2: 14%, group 1+2: 9%), χ^2^ (328) = 8.97 with *P*< .00). Recommendations to private health insurances played almost no role whatsoever (2%) (see Figure 1).

###  Clinical Decision-Making

**Figure 2:**
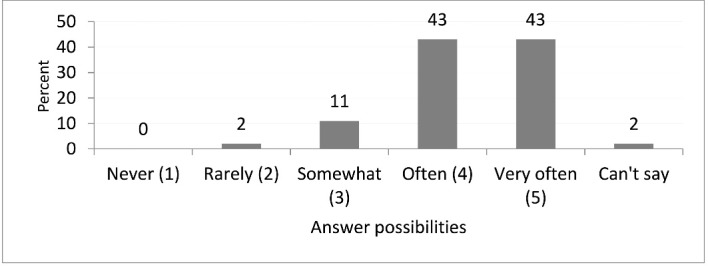
How Often the Participants Make Independent Clinical Decisions According to Their Own Assessment Without Being Limited (Group 1+2).

 A large majority of the participants, 86%, were ‘often’ and ‘very often’ able to make independent clinical decisions according to their own assessment without being limited (see Figure 2, due to rounding percentages do not sum up to 100%). Significantly more male participants stated they made independent decisions very often (*P*= .03).

 Asked to state which of a list of factors limited the participants’ own choice of diagnostic and therapeutic measures in clinical decision-making, the most limiting factor for 45% was ‘local lack of staff per patient.’ Evidence based guidelines, local ones as well as the NBHW’s national guidelines, were each experienced as limiting by 19%. The factors with the lowest influence on participants’ decisions were ‘economic incentives to undertreat,’ 7%, and ‘economic incentives to overtreat,’ 2%.

 The biggest influence (‘very much’ and ‘much’) on participants’ decision-making was for 94% their ‘own medical assessment,’ for 88% ‘guidelines in general’ and for 57% ‘a colleague’s medical assessment.’ Only 7%, assessed ‘costs for the hospital’ as influential (‘very much’ and ‘much’). The most important guidelines (‘very much’ and ‘much’) for group 1 were local guidelines (group 1: 78%), followed by ‘other national clinical guidelines/recommendations than Socialstyrelsen’s’ (group 1: 69%), and ‘Socialstyrelsen’s national guidelines’ (group 1: 67%). For group 2, the most important sources of information (‘very much’ and ‘much’) were firstly ‘European clinical guidelines’ (group 2: 85%), secondly ‘Socialstyrelsen’s national guidelines’ (group 2: 69%), and thirdly ‘local clinical guidelines’ (group 2: 69%).

###  Clinical Priority Setting

**Figure 3:**
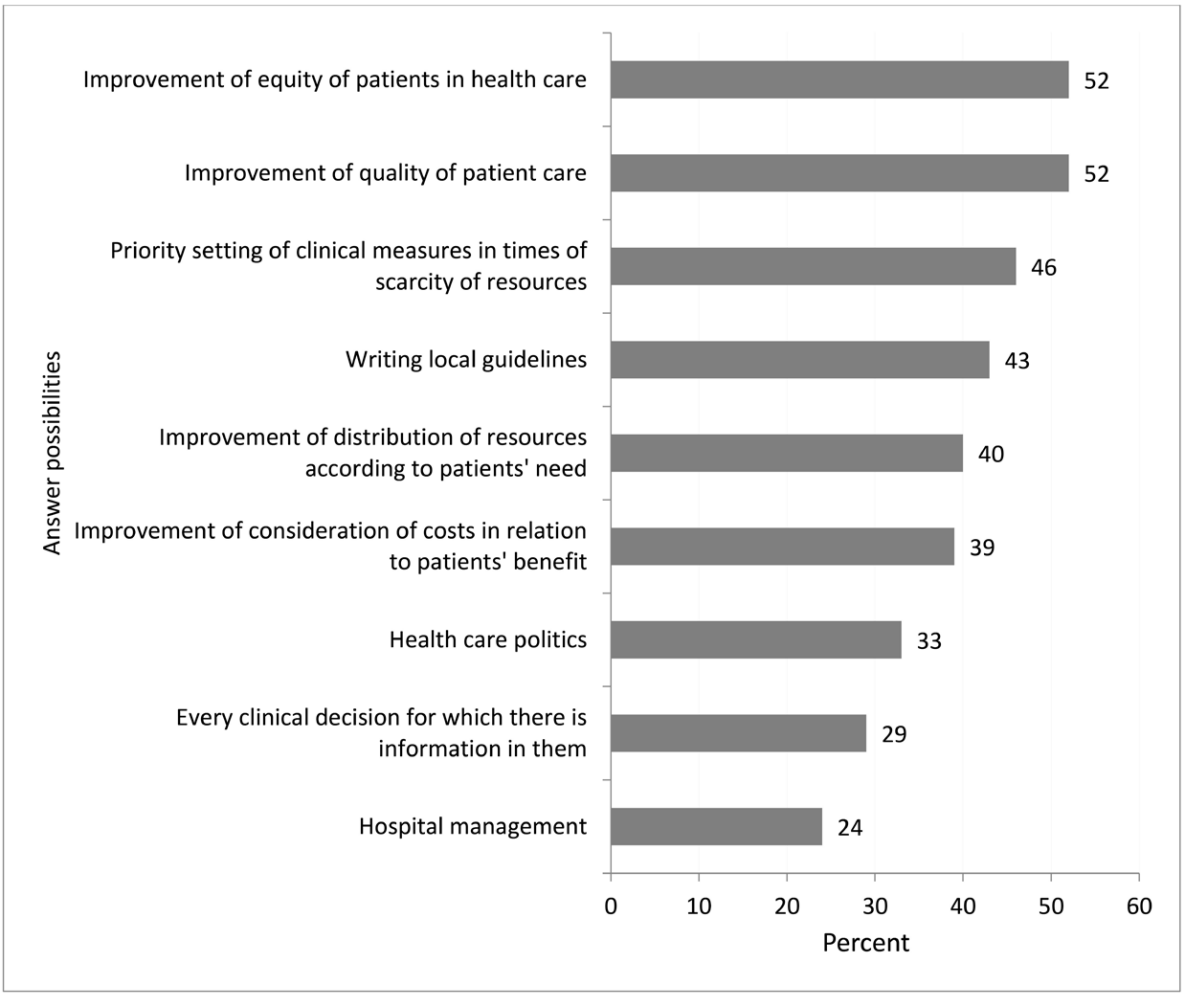
In Which Aspects Participants Consider NBHW’s National Guidelines to Be Helpful (Group 1+2). Abbreviation: NBHW, National Board of Health and Welfare.

 About one third, 36%, of the participants stated that they knew ‘much’ or ‘very much’ about the ethical platform. About two thirds, 64%, indicated to knew ‘much’ or ‘very much’ about the NBHW’s national guidelines in their area, (group 1: 50.6%, group 2: 76.5%). Regarding knowledge of the NBHW’s national guidelines, participants of group 2 were more aware of them than group 1. 11 % of group 1 chose the option ‘can’t say.’ Frequencies varied between both groups and a significant difference was shown, χ^2^ (328) = 33.10 with *P* < .00. The NBHW’s national guidelineswere considered as helpfulespeciallyfor ‘improvement of equity in healthcare’ and ‘improvement of quality of patient care’ each by 52%, for ‘priority setting of clinical measures in times of scarcity of resources’ by 46% and for ‘writing local guidelines’ by 43% (see Figure 3). Group 2 assessed the NBHW’s national guidelines as more helpful even for ‘priority setting of clinical measure in times of scarcity of resources,’ than group 1 (group 1: 38%, group 2: 53%, group 1+2: 46%, χ^2^ (328) = 7.12, *P* = .01). Over half of all participants, 57%, reported that they would like to have further knowledge and training regarding the ethical platform. Slightly fewer participants, 51%, expressed a wish for more support in the application of the NBHW’s national guidelines. Significantly more female participants asked for more support both regarding the ethical platform (*P* = .03) and the NBHW’s national guidelines (*P* = .01).

## Discussion

 As far as we are aware, this is the first study analysing whether and how clinical decision-making of physicians is influenced by the Swedish measures of priority setting. While the Swedish model is generally viewed in a favourable light, one of the concerns that was expressed in an international context was that explicit priority setting schemes might severely limit physicians in their ability to make decisions based on their own clinical experience and judgement.^10,32,33^ We wanted to examine Swedish physicians’ perception of scarcity of resources and freedom in decision-making in healthcare as well as their awareness of priority setting guidance. Our findings could be instructive for other countries considering implementing priority setting schemes in their healthcare systems.

###  Clinical Patient Care

 Rationing, described as not prescribing or performing a diagnostic or therapeutic measure during the last 6 months and not including rationing by time (waiting lists), were indicated by only 9% of the participating Swedish physicians once a day or once a week and for 44% once a month or less. By comparison, 13% of German physicians in intensive care units and cardiology stated that they ration once per day or once per week and 64% once per month or less.^27^ Physicians from Great Britain, Italy, Norway, and Switzerland rationed at a percentage of 15% once a day or once a week and 41% once a month or less.^34^ These numbers might suggest that Swedish physicians ration slightly less often than colleagues in Europe, or at least not more frequently. However, research on rationing is hampered by the fact there is usually a wish for more resources on the part of physicians, and that a tendency exists to deny and neglect scarcity.^35,36^ These factors could be at play in explaining lower frequencies. Nevertheless, only a minority, less than a tenth of all participants in our sample, reported that they restricted needed diagnostic or therapeutic measures due to scarcity of resources. This was in line with the finding on rationing.

 Coping strategies focused on communication and peer support, with discussions with colleagues and superiors identified as most important for more than half to three quarters of participants in the two groups. Approximately a third of participants reported talking to the patients about addressing scarcity. Simultaneously, more than half of all participants felt less than well-prepared to deal with scarcity in the clinical setting. These findings are in line with previous studies done when the ethical platform and the NBHW’s national guidelines were first introduced and highlight that readiness in dealing with perceived scarce resources has not improved.^15^

###  Clinical Decision-Making

 Previous political directives in Swedish healthcare aimed to provide more standardised validation by evidence-based medicine, efficiency and equity.^37^ There have however been some concerns regarding the risk that these would collide with the self-governance of the profession, its role and responsibility.^38^ Worries that ability and freedom in decision-making would be curtailed by New Public Management-associated bureaucratic actions including administrative as well as economical structures was expressed in two physicians’ appeals in Sweden.^39-41^ In addition, fears that priority setting would be limiting freedom in clinical decision-making has been expressed in other countries, too.^26^ In our study though, a large majority, 86%, of participants reported that they were ‘often’ or ‘very often’ able to make independent decisions according to their own assessment without any limitations, with male doctors stressing independence even more than female physicians. This is a relevant finding closing a gap in the literature. It is of course possible that results are due to Swedish socio-cultural aspects not transferable to other settings, but even after two decades of a priority-setting platform in place, local guidelines as well as the NBHW’s national guidelines were experienced as limiting each by only 19%. In comparison, 44% of German physicians assessed (hypothetical) guidelines that would include information about costs as a limitation of their freedom in clinical decision-making.^26^

 These findings are particularly striking in view of the fact that ‘guidelines in general’ were the second biggest influence on clinical decision-making in our sample, following ‘own medical assessment.’ Guidelines as support for evidence based medicine is highly appreciated.^42^ While the importance of guidelines is in line with other studies,^16,43^ it is nonetheless interesting to observe that even in the context of scarce resource allocation, a large majority of physicians in our sample felt largely unrestricted. In clinical life there still exist confusion about the multitude of national guidelines and recommendations,^18,44^ however, Swedish authorities plan to reduce confusion by uniting many different local and national guidelines in one place.^43^

###  Clinical Priority Setting

 The ethical platform as the foundation of Swedish priority setting was lesser known (36%) than the NBHW’s national guidelines themselves (64%). This might be seen as unexpected, since these guidelines in fact are based on best available evidence, expert views and an operationalization of the ethical platform. There was also a significant difference in knowledge, with higher knowledge in participants in cardiac care (group 2) as compared to other participants (group 1). This likely reflects that the national guideline of cardiac care was the first one ever published by NBHW and received significant attention. A previous study in the field of cardiology showed higher knowledge, about 48%, regarding the meaning of each ethical principle of the platform.^15^ A later study indicated that 90% of cardiologists/internists were very familiar with the contents and 94% positively considering the national guideline of cardiac care.^16^

 The NBHW’s national guidelines were considered in our sample as helpfulespeciallyforimproving quality and equity of patient care, achieving the aims of the ethical principles and priority setting. A similar result was found among chiefs of departments,^18^ however, until now this was not replicated in a sample with a wider range of experience and decision-making scope. Clinical relevance of the NBHW’s national guidelines was high as they were considered as helpful for ‘writing local guidelines.’

 Unsurprisingly, cardiologists (group 2) with their better awareness of the NBHW’s national guidelines applied them two to three times more often in situations of scarcity than the other participants (group 1) and considered the NBHW’s national guidelines about a third more helpful. This positive perception in line with the grade of knowledge was shown in previous studies, too.^18^

 These positive assessments could be explained by the fact that NBHW’s national guidelines for priority setting might be seen as a successful operationalization of the ethical platform. Further, and more broadly speaking, the principles underlying the ethical platform and the NBHW’s guidelines are in line with those implicitly expressed in Swedish healthcare legislation, which might lend them some democratic legitimacy. And finally, in addition, the healthcare professions in Sweden, particularly physicians, are now closely and continuously involved in developing guidelines and integrating best available evidence with ethical principles, with a large number of guidelines available in different healthcare specialities. As a part of this process, some successful method development has been carried out, eg, via collaboration between the National Centre for Priority Setting in Health Care and the NBHW.^45-47^

 In view of the wish for more knowledge, support and training about the ethical platform, expressed particularly by female physicians, it can be expected that the positive perception of the guidelines will rise further in future. Interestingly, regarding training, participants were more interested in the ethical platform than in the NBHW’s national guidelines, despite the latter being far more concrete and including an operationalization of the ethical platform. While the need for training has been found in other studies as well,^15,48,49^ more focus on the ethical principles, in response to our finding, could increase physician buy-in.

###  Limitations

 The findings must be interpreted with caution as the response rates differed between 49% in group 1 to 18% in group 2, although these are common rates for this type of research.^51,52^ The real response rate of group 2 was likely higher, as the invitation to the study probably did not reach all due to out-of-date email addresses and not all members of the Society of Cardiology were clinical physicians. However, demographic characteristics of our respondents even in group 2 corresponded with the demographic of the overall population of physicians engaged in cardiac care.^31^ Even though data gathering occurred in 2016 and 2017, results are expected to still be valid since no changes in general priority setting measures were implemented and NBHW’s national guidelines are now longstanding. The questionnaire was in English and not in the native Swedish to allow for international collaboration. The ability to communicate in English is quite high in Sweden,^53^ particularly in well-educated populations and thus we can assume that there was no significant language barrier. It cannot be fully excluded that the phrasing of questions might have led to subjective interpretations, however, pre-testing showed high consistency in the understanding and interpretation of questions. Finally, the questionnaire was quite long, which most likely had a negative impact on the response rate.

## Conclusion

 Our study has shown that the participating physicians had a fairly good awareness of the national guidelines by the NBHW including recommendations for priority setting, which are based on the Swedish ethical platform for priorities in healthcare. Compared to earlier studies, overall knowledge has declined. The NBHW’s national guidelines were especially considered to be helpful for improving equity and quality in healthcare as well as writing local guidelines. As non-binding evidence-based guidelines were assessed as very important in clinical decision-making, an implicit application of information of the NBHW’s national guidelines can be assumed. Participants who knew the NBHW’s national guidelines better, applied them more often and considered them as more helpful. The ethical principles expressed in the ethical platform as the foundation of all other priority setting measures, were lesser known and participants wished for further training.

 Even though unpreparedness of handling scarcity of resources was often reported, rationing was not reported to be more frequent than evidenced in studies from other European countries. Our findings should also assuage the worries that explicit priority setting overly restricts of physicians’ ability to make independent clinical decisions. Independence in decision-making based on own clinical assessment was perceived as high and limitations by guidelines as rare. NBHW’s national guidelines are non-binding and do offer however guidance on which interventions should be offered and which ones should not. The Swedish model can be seen as being somewhere between mandatory and voluntary.

 There are several ongoing challenges connected to the Swedish priority setting activities. First, as shown here and in previous studies, there is still a shortage of knowledge and awareness of the ethical platform among relevant decision-makers, including clinicians.^45^ There is also a lack of coordination of priority setting measures among different levels and stakeholders in the healthcare system. Furthermore, as in many other countries, in today’s Sweden different, parallel incentive structures and systems are at play, which sometimes are not fully compatible with the ethical principles expressed in the Swedish priority setting legislation. Alternative investments, concepts and logics, eg, value based medicine as well as new public management-inspired market solutions, might lead to growing health inequalities in the Swedish healthcare system and hinder the achievement of consequent, coherent and transparent priority setting.^45,50^

 That said, our study results show that it is well worth paying more attention to the many efforts of Swedish priority setting, both in Sweden as well as internationally. Priority setting guided by ethical principles can be regarded as one pathway to fair and transparent decision-making. In Sweden, the original intention based on the ethical principles should be highlighted with renewed emphasis and continuous communication within all professional levels, as well as additional training and support, should be enabled. More focus should be laid on clearer ways to present the priority setting efforts and their importance to clinicians. In addition, there are a number of open research questions. For the future we suggest investigating different stakeholders’ views on clinical priority setting, for example on fair priority setting processes, or on the reallocation and disinvestment of healthcare resources.

## Ethical issues

 Ethic approval was not applicable/not necessary for this type of study, neither in Germany (document from the ethics committee at Kiel university attached) nor in Sweden (Swedish law on ethics approval in research concerning humans, 2003:460, https://www.riksdagen.se/sv/dokument-lagar/dokument/svensk-forfattningssamling/lag-2003460-om-etikprovning-av-forskning-som_sfs-2003-460). Participation of this study was voluntary and anonymous. All participants were informed by writing.

## Competing interests

 Authors declare that they have no competing interests.

## Authors’ contributions

 AMB developed the idea of the study with CD. CD and AMB developed the study with help from BK, NE, CB, AR. CD conducted the study. CD and SM analysed the data and wrote the manuscript with help from AMB, BK, NE, CB, AR. All authors read and approved the manuscript.

## Supplementary files

Supplementary file 1 contains Table S1.
