# Supplementary material for: Clinical Priority Setting and Decision-Making in Sweden: A Cross-sectional Survey Among Physicians
Source: Int J Health Policy Manag. 2021 Mar 15;11(7):1148–57. doi: 10.34172/ijhpm.2021.16 (PMC9808196; doi:10.34172/ijhpm.2021.16)
Supplement: Supplementary file 1 — contains Table S1. [file ijhpm-11-1148-s001.pdf]

Article title: Clinical Priority Setting and Decision-Making in Sweden: A Cross-sectional Survey Among Physicians

Journal name: International Journal of Health Policy and Management (IJHPM)

Authors' information: Catharina Drees[1]*, Barbro Krevers[2,3], Niklas Ekerstad[2,3,4], Annette Rogge[1], Christoph Borzikowsky[5], Stuart McLennan[6,7], Alena M. Buyx[6]

[1]Division of Biomedical Ethics, Institute of Experimental Medicine, Christian-Albrechts-University of Kiel, Kiel, Germany.

[2]Department of Health, Medicine and Caring Sciences, Unit of Health Care Analysis, Linköping University, Linköping, Sweden.

[3]National Centre for Priorities in Health, Linköping University, Linköping, Sweden.

[4]NU Hospital Group, The Research and Development Unit, Trollhättan, Sweden.

[5]Institute of Medical Informatics and Statistics, University Hospital Schleswig-Holstein, Kiel, Germany.

[6]Institute of History and Ethics in Medicine, Technical University of Munich, Munich, Germany.

[7]Institute for Biomedical Ethics, University of Basel, Basel, Switzerland.

(*corresponding author: catharina.drees@skane.se)

Supplementary file 1

Table S1: Summary of study results of "Clinical Priority Setting and Decision-Making in Sweden"

Questions	Responses	Both groups	Group 1	Group 2
**I. CLINICAL PATIENT CARE**				
1.1. How often do the following statements match the hospital patient care that you are familiar with?				
1.1.1. All patients are equally treated.	Never (1)	0%	0%	0%
	Rarely (2)	1%	1%	1%
	Somewhat (3)	8%	4%	12%
	Often (4)	38%	36%	39%
	Very often (5)	53%	58%	48%
	Can't say	1%	1%	1%
1.1.2. All resources are distributed according to patients' need.	Never (1)	0%	0%	0%
	Rarely (2)	4%	3%	5%
	Somewhat (3)	17%	9%	25%
	Often (4)	47%	48%	45%
	Very often (5)	32%	40%	25%
	Can't say	0%	0%	1%
1.1.3. All decisions are made in consideration of costs in relation to patients' benefit.	Never (1)	0%	0%	1%
	Rarely (2)	16%	18%	13%
	Somewhat (3)	37%	33%	41%
	Often (4)	35%	38%	33%
	Very often (5)	12%	12%	11%
	Can't say	1%	0%	1%
1.1.4. All patient care is optimal.	Never (1)	0%	1%	0%

	Rarely (2)	7%	4%	10%
	Somewhat (3)	33%	30%	35%
	Often (4)	49%	56%	42%
	Very often (5)	10%	9%	11%
	Can't say	1%	1%	1%
1.2. How often did you face scarcity of resources in your clinical work during the last six months?	At least once per day	12%	9%	15%
	At least once per week	26%	27%	25%
	At least once per month	21%	25%	16%
	Less than once per month	25%	25%	26%
	Until now never	11%	7%	14%
	Can't say	5%	7%	4%
1.3. How often did you not prescribe/perform a diagnostic/ therapeutic measure because of scarcity of resources during the last six months?	At least once per day	2%	1%	2%
	At least once per week	7%	8%	7%
	At least once per month	11%	12%	9%
	Less than once per month	34%	30%	37%
	Until now never	43%	45%	41%
	Can't say	4%	4%	4%
1.4. How do you handle scarcity of resources in your clinical work? Please tick every option that matches.	I discuss the situation with my colleagues.	70%	76%	64%
	I discuss the situation with my superior.	52%	57%	47%
	I put the patient on a waiting list.	34%	36%	31%
	I talk to the patient about it.	33%	34%	31%
	I send the patient to another hospital within the "landsting".	21%	26%	15%
	I send the patient earlier home.	26%	25%	27%
	I send the patient to another department.	15%	17%	12%
	I refer to Socialstyrelsen's national guidelines.	21%	15%	28%
	I send the patient to another "landsting".	14%	12%	16%
	I do not offer all the treatment/ diagnostics some patients require.	8%	6%	9%
	I refer to the principles of the ethical platform.	9%	4%	14%
	I recommend an additional private health insurance.	2%	1%	3%
1.5 How well do you feel personally prepared to handle scarcity of resources in your clinical work?	Not at all (1)	4%	3%	4%
	Little (2)	17%	12%	21%
	Somewhat (3)	39%	44%	35%
	Well (4)	29%	32%	25%
	Very well (5)	8%	6%	10%
	Can't say	4%	3%	4%
**II CLINICAL DECISION MAKING**				
2.1 How often can you make independent clinical decisions according to your own assessment	Never (1)	0%	0%	0%
	Rarely (2)	2%	2%	1%
	Somewhat (3)	11%	7%	15%

without being limited?	Often (4)	43%	45%	41%
	Very often (5)	43%	44%	42%
	Can't say	2%	2%	1%
2.2 Which of these factors limit your own choices of diagnostic & therapeutic options in your clinical decision making? Please tick every described factor that matches.	Local lack of staff per patient	45%	53%	39%
	Political directives (e.g. "vårdgaranti", "standardiserade vårdförlopp")	27%	26%	27%
	Local lack of diagnostic/therapeutic resources	30%	25%	36%
	Administrative & bureaucratic tasks	26%	21%	30%
	Local clinical guidelines (PM)	19%	19%	19%
	Socialstyrelsen's national guidelines & their follow up	19%	15%	22%
	Directives of own clinical superior	13%	11%	15%
	Other national guidelines than Socialstyrelsen's (e.g. from Swedish clinical societies/centers)	12%	11%	13%
	Regional medication and device recommendations	17%	11%	23%
	Lack of official approvals for medical diagnostics/treatments	11%	10%	11%
	National quality registers within health care & their follow up	8%	7%	9%
	Economic incentives to undertreat	7%	4%	10%
	Economic incentives to overtreat	2%	1%	4%
2.3. How much influence do the following factors have on your own clinical decision making?				
2.3.1. Your own medical assessment	None (1)	1%	1%	1%
	Little (2)	2%	2%	2%
	Somewhat (3)	3%	4%	2%
	Much (4)	25%	22%	27%
	Very much (5)	69%	71%	68%
	Can't say	1%	1%	1%
2.3.2. Guidelines in general	None (1)	0%	0%	0%
	Little (2)	1%	1%	1%
	Somewhat (3)	11%	12%	10%
	Much (4)	48%	49%	46%
	Very much (5)	40%	37%	43%
	Can't say	0%	0%	1%
2.3.3. A colleague's medical assessment	None (1)	1%	1%	1%
	Little (2)	10%	9%	10%
	Somewhat (3)	33%	31%	35%
	Much (4)	42%	41%	42%
	Very much (5)	15%	18%	13%
	Can't say	0%	0%	1%
2.3.4. Costs for the hospital	None (1)	13%	14%	11%
	Little (2)	45%	49%	42%
	Somewhat (3)	34%	32%	36%

	Much (4)	6%	5%	7%
	Very much (5)	1%	0%	2%
	Can't say	1%	0%	2%
2.4. How much influence do the following sources of information have on your own clinical decision making?				
2.4.1. Local clinical guidelines (PM)	None (1)	1%	0%	1%
	Little (2)	5%	4%	5%
	Somewhat (3)	21%	18%	24%
	Much (4)	43%	44%	42%
	Very much (5)	31%	34%	27%
	Can't say	1%	0%	1%
2.4.2. Socialstyrelsen's national guidelines	None (1)	1%	1%	6%
	Little (2)	5%	5%	5%
	Somewhat (3)	25%	25%	25%
	Much (4)	44%	46%	42%
	Very much (5)	24%	20%	27%
	Can't say	2%	3%	1%
2.4.3. Other national clinical guidelines/ recommendations than Socialstyrelsen's (e.g. from Swedish clinical societies/centers)	None (1)	1%	3%	0%
	Little (2)	8%	6%	10%
	Somewhat (3)	22%	20%	24%
	Much (4)	47%	47%	47%
	Very much (5)	20%	22%	18%
	Can't say	2%	0%	2%
2.4.4. European clinical guidelines	None (1)	3%	7%	0%
	Little (2)	9%	17%	6%
	Somewhat (3)	23%	32%	13%
	Much (4)	38%	28%	48%
	Very much (5)	26%	15%	37%
	Can't say	1%	1%	1%
**III CLINICAL PRIORITY SETTING**				
3.1. How much do you know the ethical platform of priority setting? (The three ethical principles.)	Not at all (1)	10%	12%	9%
	Little (2)	18%	15%	21%
	Somewhat (3)	32%	33%	30%
	Well (4)	24%	25%	22%
	Very well (5)	13%	11%	14%
	Can't say	4%	4%	4%
3.2. How much do you know Socialstyrelsen's national guidelines within your own working field?(see list of existing guidelines)	Not at all (1)	3%	4%	1%
	Little (2)	8%	14%	1%
	Somewhat (3)	20%	20%	21%
	Well (4)	39%	27%	51%
	Very well (5)	25%	24%	25%
	Can't say	6%	11%	1%
3.3. In which aspects do you consider Socialstyrelsen's national guidelines to be helpful?	Improvement of equity of patients in health care	52%	54%	50%
	Improvement of quality of patient care	52%	50%	53%
	Priority setting of clinical	46%	38%	53%

	measures in times of scarcity of resources			
	Writing local guidelines	43%	47%	40%
	Improvement of distribution of resources according to patients' need	40%	41%	39%
	Improvement of consideration of costs in relation to patients' benefit	39%	35%	44%
	Health care politics	33%	31%	34%
	Every clinical decision for which there is information in them	29%	28%	29%
	Hospital management	24%	24%	24%
3.4. Would you like to get further knowledge and training in the ethical platform of priority setting?	No (0)	33%	31%	34%
	Yes (1)	57%	57%	57%
	Can't say	9%	12%	9%
3.5. Would you like to get further support for the application of Socialstyrelsen's national guidelines?	No (0)	40%	39%	41%
	Yes (1)	51%	51%	52%
	Can't say	9%	11%	7%
